# Estimate haplotype frequencies in pedigrees

**DOI:** 10.1186/1471-2105-7-S4-S5

**Published:** 2006-12-12

**Authors:** Qiangfeng Zhang, Yuzhong Zhao, Guoliang Chen, Yun Xu

**Affiliations:** 1 Department of Computer Science and Technology, University of Science and Technology of China, Hefei, Anhui, 230027, China

## Abstract

**Background:**

Haplotype analysis has gained increasing attention in the context of association studies of disease genes and drug responsivities over the last years. The potential use of haplotypes has led to the initiation of the HapMap project which is to investigate haplotype patterns in the human genome in different populations. Haplotype inference and frequency estimation are essential components of this endeavour.

**Results:**

We present a two-stage method to estimate haplotype frequencies in pedigrees, which includes haplotyping stage and estimation stage. In the haplotyping stage, we propose a linear time algorithm to determine all zero-recombinant haplotype configurations for each pedigree. In the estimation stage, we use the expectation-maximization (EM) algorithm to estimate haplotype frequencies based on these haplotype configurations. The experiments demonstrate that our method runs much faster and gives more credible estimates than other popular haplotype analysis software that discards the pedigree information.

**Conclusion:**

Our method suggests that pedigree information is of great importance in haplotype analysis. It can be used to speedup estimation process, and to improve estimation accuracy as well. The result also demonstrates that the whole haplotype configuration space can be substituted by the space of zero-recombinant haplotype configurations in haplotype frequency estimation, especially when the considered haplotype block is relatively short.

## Background

The modelling of human genetic variation is critical to the understanding of the genetic basis for complex diseases. Single nucleotide polymorphisms (SNPs) are the most frequent form of variation. The Human Genome Project and other large-scale efforts have identified millions of SNP markers. Although each marker can be analyzed independently, it is more informative to analyze them in groups. Therefore, it is useful to analyze haplotypes (haploid genotypes), which are sequences of linked markers on a single chromosome. In diploid organisms, such as human beings, chromosomes come in pairs, and experiments often yield genotype information, which blend haplotype information for chromosome pairs. There is growing evidence that, in order to better characterize the role of a candidate gene, full haplotype information should be exploited instead of using only genotype information. Unfortunately, it is both time-consuming and expensive to derive haplotype information experimentally. This explains the increasing interest in inferring haplotype information, or haplotyping, computationally. In fact, the potential use of haplotypes has led to the initiation of the HapMap project which is to investigate haplotype patterns in the human genome in different populations. Haplotype inference and frequency estimation are essential components of this endeavour.

Genotype data can be with or without any pedigree information, the first category is called population genotype data while the second one is pedigree genotype data. A large number of algorithms have been designed to estimate haplotype frequencies based on population data [1-4]. Among them, EM algorithms are most popular due to their interpretability and stability.

For any given pedigree genotype data, we can certainly discard the pedigree information and simply take the genotype sequences as the input of EM estimation algorithms for population data. However, it is well accepted that information obtained by analyzing pedigree genotype data is more reliable: the constraint provided by other members in a pedigree would force one genotype to settle on a unique haplotype pair as being most probable.

Here we propose a two-stage method to estimate haplotype frequencies in pedigrees. The first stage is the haplotyping stage, which finds out all feasible haplotype configurations for each pedigree. In the second estimation stage, we use EM algorithm to estimate haplotype frequencies in pedigrees based on the haplotype configurations inferred in the former stage.

In general, haplotyping pedigrees need consider the entire solution space of all possible consistent haplotype configurations. However, the genomic DNA can be partitioned into long blocks such that recombinations within each block are rare or even nonexistent [5,6]. Thus it is believed that haplotype configurations with fewer recombinations should be preferred in haplotype inference [7-9]. When the region of interest is so small that the expected number of recombinations in the pedigree data is very close to zero, the solution space of all consistent haplotype configurations can be replaced by that of zero recombination (provided it is non-empty) to estimate haplotype frequencies. It is because the contribution of the solutions of recombinations to the overall likelihood becomes so small compared to those of zero recombination while they bring considerable complexity to the computation. Thus, we are interested in finding the consistent haplotype configurations of zero recombination.

Wijsman [10] proposed a 20-rule algorithm, and O'Connell [11] described a genotype-elimination algorithm, both of which can be used to find out zero-recombinant haplotype configurations for pedigrees. Recently, Li and Jiang [8,9] showed that it could be solved in polynomial time. Here we propose an algorithm to find out zero-recombinant haplotype configurations in linear time using a technique called HCL-linkage analysis.

In the second stage, we use the EM algorithm to estimate haplotype frequencies based on haplotype configurations obtained from the haplotyping stage. We employ the Hardy-Weinberg Equilibrium to obtain the probabilities of founder genotypes and use a genetic model [12] to deduce the transmitted probabilities of non-founders. While the likelihood of each configuration is computed by multiplying the probabilities of each genotype, the frequency of each haplotype that appears in the configuration is calculated by a gene-counting method.

We implement all the algorithms in a C software package named HANAP (Haplotype ANAlysis in Pedigrees) and test its effectiveness and efficiency both on simulated and real data sets. The experimental results show that, our method runs much faster than the direct frequency estimation software that discards the pedigree information. Moreover, because our method utilizes such information, the estimation is more reliable.

## Methods

### Haplotyping stage: haplotyping algorithm based on HCL-linkage analysis

Excoffier's EM algorithm was widely applied in haplotype analysis [14,15]. Unfortunately, it should calculate the frequencies of all possible haplotype pairs consistent to each given genotype, which is unbearable in storage when the haplotype length grows to more than 20 [16]. O'Connell [10] showed that genetic information from relatives could be used to resolve one genotype's ambiguity, and thus reduce the number of haplotypes that should be considered. However, O'Connell's method had an exponential time complexity. Recently, Li and Jiang [8,9] showed that, for any genotype in a given pedigree, its ambiguity could be solved in cubic time (O(*m*^3^*n*^3^)), where *n *is the number of members in the pedigree and *m *is the number of loci in each genotype. Here we present a so-called HCL-linkage analysis method to do haplotyping in linear time (O(*mn*)).

#### HCL-linkage definition

Trios are simple pedigrees that contain only a pair of parents and a child. A consistent zero-recombinant haplotype configuration for a general pedigree should also be a consistent zero-recombinant haplotype configuration when restricted to each trio in this pedigree. Given trio *T *= (*F*, *M*, *C*), here *F *is the father, *M *is the mother and *C *is the child, suppose that locus *i *of *F*, *M*, and *C *have alleles {*a*, *b*}, {*c*, *d*} and {*e*, *f*} (note that {*e*, *f*} ⊂ ({*a*, *b*} ∪ {*c*, *d*})). The genotype information of *C *can be homozygous or heterozygous. If it is homozygous (*e *= *f*), then it is clear that the paternal allele and the maternal allele are the same (*e *or *f*). The situation becomes complicated if it is a heterozygous site (*e *≠ *f*). Table 1 lists out all possible situations. We can see that given the locus genotype information for the three members, it may or may not be possible to determine the paternal allele and the maternal allele for the child. We call a locus *ambiguous *if its inheritance relationship cannot be resolved.

In fact, for any trio, ignoring the ambiguous loci, the consistent (partial) haplotype configuration for the *unambiguous *loci is unique and specifies a linkage of alleles on some heterozygous loci for each node in the trio. We define such linkage as HCL-linkages (linkages of Haplotype Configuration on the non-ambiguous Loci).

##### Definition

*An ***HCL-linkage ***ψ is a quadruplet *<*v*, *RE*, *LS*, *PH*>*defined on node v and specified by the unique consistent *(*partial*) *haplotype configuration within a trio that contains v. Here v denotes the node to which the HCL-linkage belongs. LS *= {*a*_1_,...,*a*_*l*_} *is the set of heterozygous loci where the haplotype configuration has been inferred. PH *= {*ph*, *ph*'} = {(*h*_1_...*h*_*l*_), (*h*_1_'...*h*_*l*_')} *records the two *(*partial*) *haplotypes imputed on these loci. RE *= (*R*, *R*') *denotes that ph *(*respectively ph*') *is inherited from or will be passed on to the node in set R*(*R*').

An HCL-linkage describes the partial haplotype configuration of a node and the inheritance relationship between the parents and their children. Under our definition, we can conclude that every haplotype configuration should be consistent with any HCL-linkage specified by each trio in the pedigree.

#### Merge and transfer operations over HCL-linkages

In the case of multiple generations and multiple children, loci on one node may be linked by different HCL-linkages. HCL-linkages of the same node should be merged if they can. There are three cases when merging two HCL-linkages *ψ*_1 _= <*v*, (*R*_1_, *R*_1_'), *LS*_1_, {*ph*_1_, *ph*_1_'}> and *ψ*_2 _= <*v*, (*R*_2_, *R*_2_'), *LS*_2_, {*ph*_2_, *ph*_2_'}> on node *v*.

**Case (1)**: (*R*_1 _∪ *R*_1_') ∩ (*R*_2 _∪ *R*_2_') ≠ *Φ*

l.a) *R*_1 _∩ *R*_2 _≠ *Φ *or *R*_1_' ∩ *R*_2_' ≠ *Φ*, it means that both *ph*_1 _and *ph*_2 _are from the nodes in *R*_1 _and *R*_2 _said *ph*_1_' and *ph*_2_' are from the nodes in *R*_1_' and *R*_2_', so *ph*_1 _and *ph*_2 _should be on the same haplotype, and *ph*_1_' and *ph*_2_' on the other: i) *LS*_1 _∩ *LS*_2 _= *Φ*, or *LS*_1 _∩ *LS*_2 _≠ *Φ *but *ph*_1 _equals *ph*_2 _when restricted to loci in *LS*_1 _∩ *LS*_2_, it means that *ψ*_1 _and *ψ*_2 _are *compatible*. In this case, they should be merged to *ψ *= <*v*, (*R*_1 _∪ *R*_2_, *R*_1_' ∪ *R*_2_'), *LS*_1 _∪ *LS*_2_, {*ph*_1 _∪ *ph*_2_, *ph*_1_' ∪ *ph*_2_'}>, here *ph*_1 _∪ *ph*_2 _denote a longer partial haplotype, which alleles equal to those of *ph*_1 _and *ph*_2 _when restricted to loci in *LS*_1 _and *LS*_2_; ii) *LS*_1 _∩ *LS*_2 _≠ *Φ *and *ph*_1 _doesn't equal *ph*_2 _when restricted to *LS*_1 _∩ *LS*_2_, it means that *ψ*_1 _and *ψ*_2 _are *incompatible*, *i.e. *no haplotype configuration can satisfy the two HCL-linkages in the same time.

1.b) *R*_1 _∩ *R*_2_' ≠ *Φ *or *R*_1_' ∩ *R*_2 _≠ *Φ*, it means that *ph*_1 _and *ph*_2_' should be on the same haplotype, and *ph*_1_' and *ph*_2 _on the other. Similarly, *ψ*_1 _and *ψ*_2 _can be merged to *ψ *= <*v*, (*R*_1 _∪ *R*_2_', *R*_1_' ∪ *R*_2_), *LS*_1 _∪ *LS*_2_, {*ph*_1 _∪ *ph*_2_', *ph*_1_' ∪ *ph*_2_}> when they are *compatible*.

**Case (2)**: (*R*_1 _∪ *R*_1_') ∩ (*R*_2 _∪ *R*_2_') = *Φ*, but *LS*_1 _∩ *LS*_2 _≠ *Φ*,

2.a) *ph*_1 _equals *ph*_2 _(and *ph*_1_' equals *ph*_2_' consequently) or *ph*_1 _equals *ph*_2_' (then *ph*_1_' equals *ph*_2_) when restricted to *LS*_1 _∩ *LS*_2_, it means that *ψ*_1 _and *ψ*_2 _are *compatible*, in this case, they should be merged to *ψ *= <*v*, (*R*_1 _∪ *R*_2_, *R*_1_' ∪ *R*_2_'), *LS*_1 _∪ *LS*_2_, {*ph*_1 _∪ *ph*_2_, *ph*_1_' ∪ *ph*_2_'}> or *ψ *= <*v*, (*R*_1 _∪ *R*_2_', *R*_1_' ∪ *R*_2_), *LS*_1 _∪ *LS*_2_, {*ph*_1 _∪ *ph*_2_', *ph*_1_' ∪ *ph*_2_}>.

2.b) Else, *ph*_1 _doesn't equal *ph*_2 _or *ph*_2_' when restricted to *LS*_1 _∩ *LS*_2_, it means that *ψ*_1 _and *ψ*_2 _are *incompatible*.

**Case (3)**: (*R*_1 _∪ *R*_1_') ∩ (*R*_2 _∪ *R*_2_') = *Φ*, and *LS*_1 _∩ *LS*_2 _= *Φ*,

In this case, *ψ*_1 _and *ψ*_2 _cannot be merged and both should be recorded in a HCL-linkage set *Ψ*_*v *_for node *v*.

With the merge operation, we can define the normalizing of a set of HCL-linkages *Ψ*_*v*_: *normalizing *a set *Ψ*_*v *_of HCL-linkages on node *v *means repeatedly applying the merge operation for pairs of HCL-linkages in *Ψ*_*v *_until, ∀*ψ*_*i*_, *ψ*_*j *_∈ *Ψ*_*v*_, (*R*_*i *_∪ *R*_*i*_') ∩ (*R*_*i *_∪ *R*_*i*_') = *Φ*, and *LS*_1 _∩ *LS*_2 _= *Φ*. *Ψ*_*v *_is then said to be *normalized*. From now on, if there is no further notice, *Ψ*_*v *_should be normalized after any changes.

Like genetic information, HCL-linkages will be passed on from generations to generations. Without loss of generality, let us define the transfer of HCL-linkage information from child *C *to its parent *F*. The other case from *F *to *C *would be similar. Let *Ψ*_*C *_and *Ψ*_*F *_represent the normalized HCL-linkage sets of *C *and *F *respectively, and let *HS *be the set of homozygous loci of *F*. The transfer of *Ψ*_*C *_from *C *to *F *results in changes to *Ψ*_*F*_, where each *ψ*_*C *_= <*C*, (*R*_*C*_, *R*_*C*_'), *LS*_*C*_, {*ph*_*C*_, *ph*_*C*_'}> ∈ *Ψ*_*C *_is transferred independently. There are two cases to consider.

**Case (1)**: if *F *∈ *R*_*C *_(or respectively, *F *∈ *R*_*C*_'), add *ψ*_*F *_= <*F*, ({*C*}, *Φ*), *LS*_*C *_- *HS*, {*ph*_*F*_, *ph*_*F*_'}> to *Ψ*_*F*_, here *ph*_*F *_equals the resulting partial haplotypes of *ph*_*C *_(respectively *ph*_*C*_') when restricted to loci in *LS*_*C *_- *HS *and *ph*_*F*_' is the compensatory partial haplotypes of *ph*_*F *_consistent to genotype *g*_*F*_.

**Case (2)**: else, *F *∉ *R*_*C *_∪ *R*_*C*_': i) both *ph*_*C *_and *ph*_*C*_' are consistent with the partial genotype *g*_*F *_when restricted to loci in *LS*_*C*_, then add *ψ*_*F *_= <*F*, (*Φ*, *Φ*), *LS*_*C *_- *HS*, {*ph*_*F*_, *ph*_*F*_'}> to *Ψ*_*F*_, here *ph*_*F *_and *ph*_*F*_' equal the resulting partial haplotypes *of ph*_*C *_and *ph*_*C*_' when restricted to loci in *LS*_*C *_- *HS*; ii) *ph*_*C*_' (respectively *ph*_*C*_) is not consistent with the partial genotype *g*_*F *_when restricted to loci in *LS*_*C*_, then add *ψ*_*F *_= <*F*, ({*C*}, *Φ*), *LS*_*C *_- *HS*, {*ph*_*F*_, *ph*_*F*_'}> to *Ψ*_*F*_, here *ph*_*F *_equals the resulting partial haplotypes of *ph*_*C *_(respectively *ph*_*C*_') when restricted to loci in *LS*_*C *_- *HS *and *ph*_*F*_' is the compensatory partial haplotypes of *ph*_*F *_consistent to genotype *g*_*F*_. Note that at least one of *ph*_*C *_and *ph*_*C*_' should be consistent with the partial genotype *g*_*F*_.

Remember that *Ψ*_*F *_should be normalized whenever adding a new HCL-linkage to it. In the case of transferring an HCL-linkage *ψ*_*F *_from *F *to *C*, resulting in adding *ψ*_*C *_= <*C*, (*R*_*C*_, *R*_*C*_'), *LS*_*C*_, {*ph*_*C*_, *ph*_*C*_'}> to *Ψ*_*C*_, note that we should add *M *into *R*_*C*_' whenever we have determined that *F *∈ *R*_*C*_.

Our merge and transfer operations will not bring more or lose any HCL-linkage information for building consistent haplotype configurations.

#### Main HCL-linkages analysis haplotyping algorithm

Before the algorithm, we preprocess each trio in the pedigree. Whenever a trio specifies an HCL-linkage for node *v*, it will be stored in the HCL-linkage set *Ψ*_*v*_. The objective of the algorithm is to collect the complete HCL-linkage information for each node, which is accomplished by traversing the tree twice.

Firstly, we will convert the input pedigree into a rooted searching tree **T **(at an arbitrary node *R*) (Step 1). Then we traverse **T **in post-order to transfer and merge the HCL-linkage information for each node from its relatives (Step 2). We do this from the left lowest nuclear family **F**_o_. The HCL-linkages in nuclear family **F**_o _will be merged at both parents, and then be transferred to the root of the sub-tree. The same operations will be conducted in its parental nuclear family on HCL-linkages specified in this family as well as on those transferred from its child families. And at last, we collect all the HCL-linkages at the root *R*. In Step 3, we traverse **T **again in pre-order and transfer the linkage in another direction from *R *to its farmost descendants.

After step 3, the HCL-linkage set of each node preserves all HCL-linkages in the pedigree. In step 4, we choose a node *v *arbitrarily. Set *Ψ*_*v *_contains several HCL-linkages *ψ*_1_, *ψ*_2_,...,*ψ*_*l *_defined on disjoint locus set *LS*_1_, *LS*_2_,...*LS*_*l*_. When a set of loci are linked by one HCL-linkage, they can be viewed as a compound locus, and the two partial haplotypes can be viewed as two compound alleles. These "loci" (and "alleles") will be treated equally as the other heterozygous loci and homozygous loci that are not involved in any HCL-linkage. We arbitrarily select one allele from the two at each locus to form a haplotype; the other alleles form another haplotype. It is called an *imputing schema*. Whenever the haplotype configuration of one node is determined, it can be used to determine the configurations of its relatives, and those of the whole pedigree at last.

**Figure 1:**
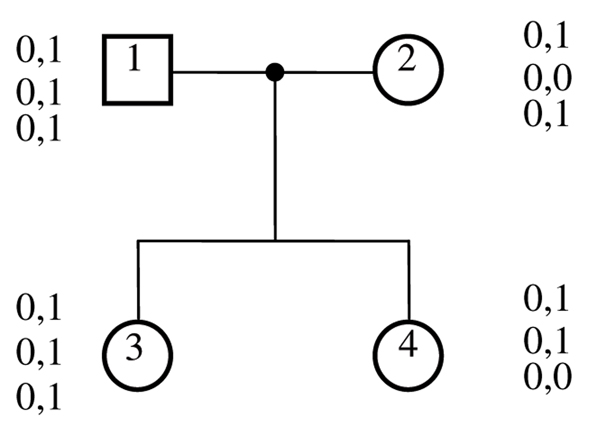
Incompatibleness when imputing alleles.

During our algorithm, Incompatibleness may occur when normalizing HCL-linkage set *Ψ*_*v*_. Then we declare that there is no solution and exit from the algorithm immediately. Even in step 4, incompatibleness may still occur when applying the haplotypes of the parents to resolve the genotype of the children in the case that an individual node has multiple children. Figure 1 shows an example. The key point is, if it exists a consistent haplotype configuration for a nuclear family (*F*, *M*, *C*_1_, *C*_2_,...,*C*_*d*_), every arbitrary imputing schema *s *can output one feasible solution *ζ*. Contrarily, if one imputing schema ends with incompatibleness, other schemata will fail too. We will prove this in the appendix.

The time complexity and space complexity of our algorithm are both *O*(*mn*) where *n *is the number of the members in the pedigree and *m *is the length of the loci.

### Frequency estimation stage

Suppose that we are given *K *pedigrees **P **= {*P*_1_, *P*_2_,...,*P*_*K*_}. Each *P*_*i *_consists of *n*_*i *_nodes *v*_*i,j *_(1 ≤ *i *≤ *K*, 1 ≤ *j *≤ *n*_*i*_), in which the first *n*_*i*_' are founders. The genotype of node *v*_*i,j *_(1 ≤ *j *≤ *n*_*i*_) is *g*_*i,j*_. Suppose that there are *π*_*i *_consistent solutions for pedigree *P*_*i *_and the *s*-th solution is: *S*_*s,i *_= <*S*_*s,i*,1_, *S*_*s,i*,2_,...,> (1 ≤ *s *≤ *π*_*i*_), where *S*_*s,i,j *_= <*α*_*s,i,j*,1_, *β*_*s,i,j*,2_> is a haplotype pair of genotype *g*_*i,j*_. All haplotypes appear in these solutions form a list of haplotypes *H *= {*h*_1_, *h*_2_,...,*h*_*l*_*} *with frequencies Θ = {*θ*_1_, *θ*_2_,...,*θ*_*l*_}, here *θ*_1 _+ *θ*_2 _+ ... + *θ*_*t *_= 1 is what we want to estimate.

The likelihood of haplotype frequencies given the observed pedigree data is,



Under the assumption of random mating, the paternal haplotype configuration and the maternal haplotype configuration are independent, and the child's haplotype configuration is transmitted from its parents. We have:



Here Pr (*S*_*s,i,j*_|Θ) is the probability of haplotype configuration of the founder nodes, it can be computed using the Hardy-Weinberg Equilibrium. Pr(*S*_*s,i,j*'_|<, >) is the gamete transmission probabilities of haplotype configuration *S*_*s,i,j *_with the parental haplotype configurations of  and . It can be computed using a genetic model presented by Elston and Stewart [6].

EM algorithm estimates the haplotype frequencies Θ starting with the initial arbitrary values Θ^(0) ^= {*θ*_1_^(0)^, *θ*_2_^(0)^,...,*θ*_*l*_^(0)^}. These initial values are used as if they were the unknown true frequencies to estimate solution frequencies Pr(*S*_*s,i*_|Θ) (the expectation step). These expected solution frequencies are used in turn to estimate haplotype frequencies at the next iteration Θ^(1) ^= {*θ*i^(1)^, *θ*_2_^(1)^,...,*θ*^(1)^} (the maximization step), and so on, until convergence is reached.

Suppose that in the *r*-th iteration, Θ = Θ^(*r*) ^and we want to estimate Θ^(*r*+1)^. Then we have:



Let *δ*_*i,j,t *_be an indicator variable equalling the number of haplotype *h*_*t *_appear in solution *S*_*s,i*_. Then the haplotype frequencies can be computed using a gene-counting method,



There are several ways to initialize the haplotype frequencies Θ = {*θ*_1_, *θ*_2_,...,*θ*_*l*_}. For instance, the initial haplotype frequencies can be chosen at random, or all haplotypes are equally frequent, *i.e. *Θ_*t*_^(0) ^= 1/*l *(*t *= 1, 2,...,*l*). Or that all initial haplotype frequencies are equal to the product of the corresponding single-locus allele frequencies (*i.e.*, a complete linkage equilibrium). Also, we can set all feasible solutions for each pedigree to be equally likely, *i.e. *Pr(*S*_*s,i*_|Θ^(0)^) = 1/*π*_*i*_, (*j *= 1,2,...,*π*_*i*_). We can even initialize the haplotype frequencies by counting their occurrence in all the feasible solutions. Since in practical applications the EM algorithm could be trapped in some local maximum, we recommend to restart the algorithm several times with different initial haplotype frequencies and better with a randomized additive perturbation.

The stopping (convergence) criterion is defined as the absolute value of the difference of Θ between consecutive iterations being less than some small value *ε *> 0.

## Results

### Simulated data set

In order to generate a pedigree genotype data set for simulation experiments, we generate a population of haplotypes H* first, where each locus of each haplotype is set to some allele according to the probability distribution function *P*. In our simulation, we generated haplotypes of SNP loci as well as haplotypes of micro-satellite loci. For a biallelic SNP locus *i*, suppose that *i *happens to be one state with a probability of *p*_*i*_, and to be the other state with a probability of (1 - *p*_*i*_). For a micro-satellite loci, suppose it has w different alleles: *a*_1_, *a*_2_,...,*a*_*w*_, each appears with the probability of *p*_1_, *p*_2_,...,*p*_*w *_(*p*_1 _+ *p*_2 _+...+ *p*_*w *_= 1).

Each founder node in any tested pedigree is arbitrarily assigned a pair of haplotypes according to their frequencies θ*. The two haplotypes of a non-founder node are arbitrarily selected from those of its parents (one from the father, one from the mother). At last, the pair of haplotypes of the same node is blended to form a genotype corresponding to that node.

All experiments are conducted on a Windows server with 1.7G Hz CPU and 256 MB RAM. And for each parameter setting, 100 copies are randomly generated and the performance is evaluated by computing the average numbers in these 100 runs.

### Running time of the haplotyping algorithm

One of the main contributions of our paper is to do haplotyping in linear time, so we firstly examine the running time with respect to different number of nodes of each pedigree (*n*) and different number of loci in each sequence (*m*).

**Figure 2:**
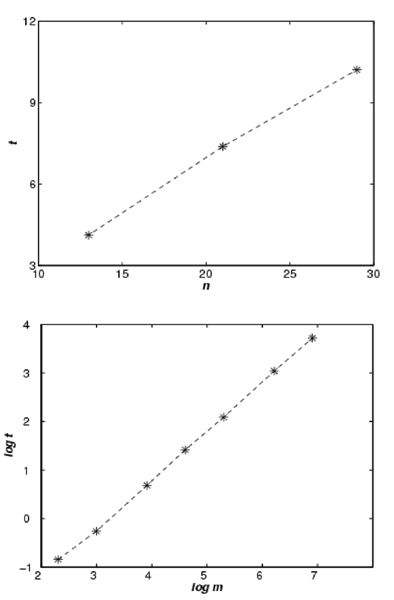
Running time of the haplotyping algorithm: (a) running time *v*s. number of nodes (*m *= 100); (b) running time *v*s. number of loci (*n *= 13).

Several different tree pedigree structures are used in the simulation, the first pedigree is Figure 1 in [15], which is a tree with 13 nodes. The second one is Figure 8 in [9], which is a tree with 29 nodes. The third one is a 21 node pedigree from Figure 5 of [15]. The results are given in Figure 2. It is obvious that our HCL-analysis haplotyping algorithm runs in linear time and thus could be applied to large-scale haplotype analysis.

### Number of solutions

We compare the numbers of haplotypes that should be considered in the estimation stage, with and without the haplotyping stage. In our experiment, we set *P*_1 _(*p*_1 _= *p*_2 _= 0.5) and *P*_2 _(*p*_1 _= 0.9, *p*_2 _= 0.1) for SNP loci, and set *w *= 4, *P*_3 _(*p*_1 _= *p*_2 _= *p*_3 _= *p*_4 _= 0.25) and *P*_4 _(*p*_1 _= 0.5, *p*_2 _= *p*_3 _= 0.2, *p*_4 _= 0.1) for micro-satellite loci. We let |H*| = 20, and *θ*_1_* = 0.2, *θ*_2_* = *θ*_3_* = *θ*_4_* = 0.1, *θ*_5_* = *θ*_6_* = *θ*_7_* = *θ*_8_* = 0.05, *θ*_9_* = *θ*_10_* = ... = *θ*_20_* = 0.025.

When only trio pedigrees are considered, the average numbers of haplotypes are recorded in Table 2. We can see from the table that the numbers of haplotypes that should be estimated have been greatly reduced after the haplotyping stage (HANAP *vs. *directly), which will immediately bring the improvement on the running time.

We also consider a more complex pedigree that contains 13 nodes (Figure 1 of [15]). The average numbers of haplotypes are recorded in Table 3. We find that the number of haplotypes that should be estimated is even much smaller. We also notice that the number of haplotypes is growing with the length of haplotypes and the number of pedigrees. However, it grows very slowly.

### Running time of HANAP

EM-DeCODER is a popular software using the EM algorithm to estimate the haplotype frequencies based on population data. As we have pointed out, it can be used to estimate haplotype frequencies in pedigrees, simply by discarding the pedigree information. Here we also compare the running times of HANAP and EM-DeCODER.

**Figure 3:**
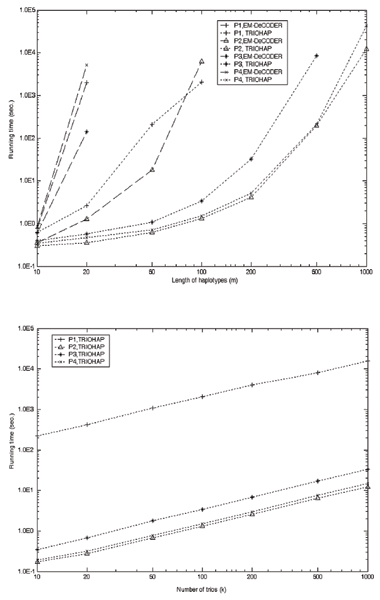
The running time of HANAP and EM-DeCODER: (a) running time *v*s. length of haplotypes (*K *= 100); (b) running time *v*s. number of trios (*m *= 100).

Figure 3 shows their running times over different number of trios (*k*), length of haplotypes (*m*) and distributions of allele-probability (*P*). We can learn from the figure that HANAP runs much quicker than EM-DeCODER, and thus can be applied to much larger instances. We also notice that the running time of both HANAP and EM-DeCODER increase exponentially with the length of haplotypes while increasing near-linearly with the number of trios (the running time of EM-DeCODER is not plotted in Figure 3(b) because haplotypes of length 100 are out of its capability).

### Accuracy rate of HANAP

**Figure 4:**
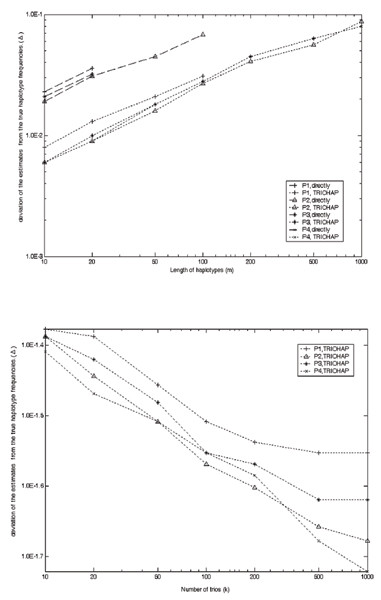
The estimate deviation of HANAP and EM-DeCODER: (a) deviation *v*s. length of haplotypes (*K *= 100); (b) deviation vs. number of trios (*m *= 100).

We define a parameter Δ to incarnate the deviation of the estimate haplotype frequencies from the underlying ones. Because the simulation data are generated according to the Θ*, we recognize that as the underlying true frequencies. Suppose the estimate haplotype set is *H*^E ^with frequencies Θ^E^. Compare *H*^E ^with H*. Suppose the estimate frequencies of the 20 haplotypes in H* are *θ*_1_^E^, *θ*_2_^E^,...,*θ*_20_^E^. We let,



Figure 4 shows the deviation of the estimate of HANAP and EM-DeCODER over different number of trios (*k*), length of haplotypes (*m*) and distributions of allele-probability (*P*). We can learn from the figure that the deviation of HANAP is smaller than that of EM-DeCODER, which means HANAP is more accurate. We have also noticed that the deviation of the estimate increases with the length of haplotypes, and decreases with the number of trios.

### Two real data sets

We also test the efficiency and accuracy of HANAP on two real data sets. The first real data set is from dbMHC|ABDR, a set of 122 trios. Each genotype of these trios contains 31 markers of the same position on chromosome 6, 10 of which are micro-satellite markers and others are SNPs. We run HANAP to find the most frequent haplotypes (with frequencies larger than 0.01). A list of 20 haplotypes is found by HANAP. Their frequencies are shown in Table 4. It only takes HANAP 0.97 second to find these haplotypes while it is out of the capability of EM-DeCODER.

The second data set is from the CEPH database [17], which contains 65 families; each consists of only three generations, usually with four grandparents, two parents and a number of children. Figure 5 in [15] shows a typical family with 21 nodes.

A great portion of the alleles in this data set have not been identified, and will be viewed as missing data. We carefully selected a data set of 28 families (totally 482 nodes) on a block (48 markers) from chromosome 14 (452 markers in total) with no recombination. Both HANAP and PHASE (another widely used software package [3] based on the GS algorithm) are applied to this data set. HANAP inferred 36 haplotypes with frequency larger than 0.01 and PHASE inferred 39 ones, among which 31 are common. Although we are not sure which output is closer to the real cases, the running time of HANAP (13*m*24*s*) is extremely shorter than that of PHASE (21*h*14*m*).

## Discussion

### Complexities of the HCL-linkage analysis haplotyping algorithm

We show that the algorithm runs in *O*(*mn*) time and *O*(*mn*) space. The pre-process need calculate no more than 3*n *HCL-linkages in no more than *n *trios. Each HCL-linkage can be computed in *O*(*m*) time, so the pre-process can be done in *O*(*mn*) time. It takes step 1 *O*(*n*) time to construct the rooted tree. In step 2 and step 3, we have to traverse the whole tree, and visit each node for no more than constant times.

When we process the HCL-linkages from the left lowest nuclear family, we should merge the *d*_1 _HCL-linkages at each parent node (if we can), it need *O*(*d*_1_*m*) time, here *d*_1 _is the number of children in this family. We need another *O*(*d*_1_*m*) time to exchange the HCL-linkage information between the two parents and transfer that to its root *R*_1 _in the search tree **T**. So we need *O*(*d*_1_*m*) time in total to process this nuclear family. When we transfer the normalized HCL-linkage set  = {*ψ*_1_, *ψ*_2_,...*ψ*_*k*_} to the upper nuclear families, we only need to remember that all *ψ*_*i *_is coming from *R*_1_, so for each *ψ*_*i*_, it will only take *O*(1) time to process *RE*_*i*_, and *O*(|*LS*_*i*_|) time to process *LS*_*i *_and *PH*_*i*_. The summation time is no more than *O*(*k *+ *LS*_1 _+ *LS*_2 _+...+ *LS*_*k*_) = *O*(*m*) because *LS*_*i *_are disjoint subsets of {1,2,...,*m*}. In other words, the HCL-linkages in one nuclear family won't increase the processing time of its adjacent families. So the total running time to process all nuclear familes is no more than *O*(*d*_1_*m *+ *d*_2_*m *+...+ *d*_*x*_*m*) = *O*(*nm*), here *x *is the number of nuclear families in the whole pedigree.

We need another *O*(*mn*) time to complete step 4. Therefore, the time complexity of this algorithm is *O*(*mn*).

For the computation, we need to maintain a data structure to store the HCL-linkage set *Ψ*_*v *_for each node *v*; we can maintain the storage always below *O*(*d*_*i*_*m*) for nuclear family **F**_*i*_. So the space complexity of the algorithm is also *O*(*d*_1_*m *+ *d*_2_*m *+...+ *d*_*x*_*m*) = *O*(*nm*).

### Effectiveness of the haplotyping phase

Excoffier used the EM algorithm to estimate haplotype frequencies while ignoring the pedigree information. Here we adopt a two-stage method, which tries to reduce the number of possible haplotypes to be considered in the stage of estimation by utilizing the relatives' information to do haplotyping at first.

Suppose we are estimating haplotype frequencies in trios and each haplotype consists of *m *biallelic SNP loci. For locus *i*, suppose that *i *happens to be one state with a probability of *p*_*i*_, and to be the other state with a probability of (1 - *p*_*i*_). Then locus *i *of the genotype is heterozygous with the probability of 2*p*_*i*_(1 - *p*_*i*_). Suppose the expected value of *p*_*i *_is *p*, then the genotype is expected to have 2*p*(1 - *p*)·*m *heterozygous loci. As a consequence, a total number of 2^2*p*(1-*p*)·*m*-1 ^possible haplotype pairs is expected to be considered if we use the EM algorithm directly. However, the probability that locus *i *in a trio is ambiguous is 2*p*_*i*_(1 - *p*_*i*_)·2*p*_*i*_(1 - *p*_*i*_)·2/4 = 2*p*_*i*_^2^(1 - *p*_*i*_)^2^. So the expected number of possible haplotype configurations for the trio is . If *p *= 1/2, our method can handle *λ *= (2*p*(1 - *p*))/(2*p*^2^(1 - *p*)^2^) = 4 times longer genotypes than Excoffier's methods. Moreover, in most cases, the more frequent allele at one locus appears with a probability of more than 0.9, so our method usually can handle *λ *= 1/*p*(1 - *p*) > 10 times longer genotypes.

Furthermore, if each locus of the haplotype is a micro-satellite locus, and it has *l *different alleles: *a*_1_, *a*_2_,...,*a*_*l*_, each appears with the probability of *p*_1_, *p*_2_,...,*p*_*l*_. then the expected number of possible haplotype pairs for a genotype is , the expected number of feasible haplotype configurations for a trio is , so our method usually can handle  times longer genotypes. For example, when *l *= 8, and *p*_1 _= *p*_2 _= ... = *p*_8 _= 1/8, *λ *= 64, *i.e. *our method can be applied to cases of much larger scale.

## Conclusion

We present a two-stage method to do haplotyping and to estimate haplotype frequencies for pedigree genotype data in this paper. Given a set of pedigrees, it firstly determines all feasible haplotype configurations for each pedigree, then uses the EM algorithm to estimate the haplotype frequencies based on the inferred haplotype configurations. Because a large number of illegal haplotypes have been eliminated from the possible haplotype list, our method is both more efficient and more accurate. The experimental results show that, HANAP runs much faster than EM-DeCODER, and thus can be applied to much larger scale of instances. Moreover, the deviation of the estimate of HANAP is smaller than that of EM-DeCODER, which means it is more accurate.

Our method suggests that pedigree information is of great importance in haplotype analysis. It can be used to speedup estimation process, and to improve estimation accuracy as well. The result also demonstrates that whole haplotype configuration space can be substitute by the space of zero-recombinant haplotype configurations in haplotype frequency estimation, especially when the considered haplotype block is relatively short.

## Authors' contributions

QZ proposed the whole estimation framework and both the haplotyping and the estimation algorithms, and wrote the manuscript. YZ implemented the software and helped writing the manuscript. GC and YX participated to the design of the study and wrote the manuscript. All authors read and approved the final manuscript.

## Appendix

### Correctness of the HCL-linkage analysis haplotyping algorithm

First, we point out that every consistent haplotype configuration for pedigree *P *should be consistent with all HCL-linkages calculated by the unique partial solutions within trios, and our merge and transfer operations keep this feature during the whole process, *i.e. *if *ζ *is a haplotype configuration for *P*, and *ζ *is consistent with all the HCL-linkages in the pedigree, it should also be consistent with those after the merge and transfer operations.

Obviously, HCL-linkages are sufficient to generate a consistent haplotype configuration within a trio. We prove that:

#### Lemma

*If it exists consistent haplotype configuration for a nuclear family *(*F*, *M*, *C*_1_, *C*_2_,...,*C*_*d*_)*, every imputing schema s of arbitrarily imputing the *(*compound*) *alleles at one node can output one feasible solution ζ. Contrarily, if one imputing schema ends with incompatibleness, there is no consistent haplotype configuration*.

##### Proof

Firstly, HCL-linkages are necessary for constructing the consistent haplotype configurations, which means that if there is a feasible solution, it should correspond to one imputing schema.

Secondly, we show that if one imputing schema outputs a feasible solution, all the schemas output feasible solutions.

In particular, for a trio (*F*, *M*, *C*_1_), without loss of generality, we suppose that Step 4 starts by imputing the "alleles" of node *F*. For a specified "locus" *i*, the two alleles of *F*, *M *and *C*_1 _are denoted as (*a*, *b*), (*c*, *d*) and (*e*, *f*), and the partial haplotypes from locus 1 to locus (*i *- 1) are denoted as (*ph*_1_, *ph*_2_), (*ph*_3_, *ph*_4_) and (*ph*_5_, *ph*_6_).

Suppose in schema *s*, allele *a *is imputed to *ph*_1_, denoted as *ph*_1 _← *a*, and *ph*_2 _← *b*, *ph*_3 _← *c*, *ph*_4 _← *d*, *ph*_5 _← *e*, *ph*_6 _← *f*. If *s *outputs a consistent haplotype configuration for trio (*F*, *M*, *C*), we proof that *s*': *ph*_2 _← *a*, *ph*_1 _← *b*, *ph*_4 _← *c*, *ph*_3 _← *d*, *ph*_6 _← *e*, *ph*_5 _← *f *outputs another consistent haplotype configuration.

The loci from 1 to (*i *- 1) can be viewed as a compound locus *I*. Let's refer to Table 1, because we can impute *a *or *b *to *ph*_1_, either or both of "locus" *I *and *i *should be *ambiguous*. Else, they will be linked to a bigger compound "locus" in Step 2 or 3 by HCL-linkages. Whatever *a *or *b *will be linked with *ph*_1_, we can not impute that arbitrarily in Step 4. Without loss of generality, we assume that locus *i *is ambiguous, which means that *a *≠ *b*, and {*a*, *b*} = {*c*, *d*} = {*e*, *f*} (please refer to Table 1). We can prove that *s*' is also a consistent haplotype configuration by enumeration. Because nuclear family (*F*, *M*, *C*_1_, *C*_2_,...,*C*_*d*_) is the intersection of trio (*F*, *M*, *C*_1_), (*F*, *M*, *C*_2_),...,(*F*, *M*, *C*_*d*_), the above prove shows that both both *s*: *ph*_1 _← *a*, *ph*_2 _← *b*, *ph*_3 _← *c*, *ph*_4 _← *d *and *s*': *ph*_2 _← *a*, *ph*_1 _← *b*, *ph*_4 _← *c*, *ph*_3 _← *d *will lead to a consistent haplotype configuration for the family.

We call *s *to *s*' a walk step by switching *ph*_1 _← *a *to *ph*_1 _← *b*. Obviously, for any two imputing schemata *s*_1 _and *s*_2_, we can transfer *s*_1 _to *s*_2 _by consecutive walk steps. So *s*_1_and *s*_2 _will lead to a consistent haplotype configuration or neither can.

This lemma indicates that our algorithm works in a nuclear family. We now can prove the correctness of our HCL-linkage analysis haplotyping algorithm by induction, *i.e. *if there is at least one feasible solution for a general pedigree, HCL-linkages are sufficient to generate all solutions.

Suppose that the root *R *has multiple child mating nodes: *O*_1_,...,*O*_*r*_, each represents a nuclear family; and the algorithm works in all sub-trees, *i.e. *all of the feasible solutions for those sub-trees can be directly deduced from the HCL-linkages collected from the sub-trees and stored at their roots. From the former lemma, we know that if incompatibleness of type II occurs, there is no feasible solution. We assume that there is no incompatibleness of type II and there are always feasible solutions for all sub-trees. Suppose that haplotype configuration *ζ *is consistent with all the HCL-linkages at root *R *(and all the HCL-linkages in the *P *consequently). Then *ζ *should be consistent haplotype configuration when restricted to any nuclear family of *O*_1_,...,*O*_*r*_, and it should also be consistent with the HCL-linkages at the root of lower sub-trees of *O*_1_,...,*O*_*r*_. By induction, *ζ *should be a feasible solution when restricted to any of these sub-trees. So *ζ *is a consistent haplotype configuration of the whole pedigree *P*.

## Figures and Tables

**Table 1:** Imputing the paternal allele and the maternal allele for the child at a single locus

Conditions	Paternal	Maternal
*e *= *f*	*e*	*f*
*e *≠ *f*, *a *= *b*		
*e *= *a*	*e*	*f*
*f *= *a*	*f*	*e*
*e *≠ *f*, *a *≠ *b*, *c *= *d*		
*e *= *c*	*f*	*e*
*f *= *c*	*e*	*f*
*e *≠ *f*, *a *≠ *b*, *c *≠ *d*		
*a *= *c*, *b *= *d*	*Ambiguous*	
*a *= *c*, *b *≠ *d*		
*e *= *b or f *= *d*	*e*	*f*
*e *= *d or f *= *b*	*f*	*e*
*a *≠ *c*, *b *= *d*		
*e *= *a or f *= *c*	*e*	*f*
*e *= *c or f *= *a*	*f*	*e*
*a *= *d*, *b *= *c*	*Ambiguous*	
*a *= *d*, *b *≠ *c*		
*e *= *b or f *= *c*	*e*	*f*
*e *= *c or f *= *b*	*f*	*e*
*a *≠ *d*, *b *= *c*		
*e *= *a or f *= *d*	*e*	*f*
*e *= *d or f *= *a*	*f*	*e*
*a *≠ *c *≠ *b *≠ *d*		
*e *= *a or e *= *b*	*e*	*f*
*e *= *c or e *= *d*	*f*	*e*

**Table 2:** Comparison of number of haplotypes (|*H*|) on trio pedigrees

Parameter settings (*m*, *k*)	directly				HANAP			
	
	*P*_1_	*P*_2_	*P*_3_	P_4_	P_1_	P_2_	*P*_3_	*P*_4_
(20, 20)	3.18e4	3.43e2	2.06e6	1.02e6	5.83e2	72.2	1.12e2	76.4
(20, 100)	1.49e5	1.68e3	1.02e7	5.02e6	2.67e3	3.55e2	5.72e2	3.61e2
(20, 200)	2.56e5	3.30e3	2.02e7	0.98e7	4.45e3	5.96e2	1.14e3	6.02e2
(100, 20)	N/A	8.13e6	N/A	N/A	5.90e5	2.60e2	6.91e2	2.74e2
(100, 100)	N/A	1.62e7	N/A	N/A	2.68e6	1.31e3	3.42e3	1.52e3
(100, 200)	N/A	3.21e7	N/A	N/A	4.65e6	2.55e3	6.91e3	2.89e3
(200, 20)	N/A	N/A	N/A	N/A	N/A	7.92e2	6.08e3	1.07e3
(200, 100)	N/A	N/A	N/A	N/A	N/A	4.09e3	3.21e4	5.17e3
(200, 200)	N/A	N/A	N/A	N/A	N/A	7.79e3	6.22e4	1.03e4

**Table 3:** Comparison of number of haplotypes (|*H*|) on a general pedigree

Parameter settings (*m*, *k*)	directly				HANAP			
	
	*P*_1_	*P*_2_	P_3_	*P*_4_	*P*_1_	*P*_2_	*P*_3_	*P*_4_
(20, 20)	2.31e5	1.07e3	3.65e6	1.65e6	20.78	18.34	20.15	20.05
(20, 50)	2.53e5	2.38e3	6.82e6	2.24e6	21.0	19.39	21.2	20.67
(20, 100)	2.96e5	4.51e3	1.08e7	3.38e6	20.9	19.5	20.75	20.8
(200, 20)	N/A	N/A	N/A	N/A	26.8	21.3	22.1	22.4
(200, 50)	N/A	N/A	N/A	N/A	34.4	22.1	23.5	24.2
(200, 100)	N/A	N/A	N/A	N/A	54.7	23.9	28.9	32.8
(500, 20)	N/A	N/A	N/A	N/A	1.41e2	22.4	23.2	23.9
(500, 50)	N/A	N/A	N/A	N/A	2.24e2	29.5	35.7	30.7
(500, 100)	N/A	N/A	N/A	N/A	4.06e2	30.1	41.4	42.5

**Table 4:** The frequencies of the 20 most frequent haplotypes found by HANAP

Id	Alleles of Marker Set	*θ*
1	CCAGGTAGCGCGAAGCATTTCTGTAGTACGA	0.037
2	CCAGGTAGCGCTCTAAGCAACTGGCGACGAG	0.043
3	CCCGGTGGTACGAAGCACAATCGGCGAACAC	0.025
4	CCCGGTGGTACGAAGCACAATCGTAGTACGA	0.102
5	CCCGGTGGTACGAGGTATCTCTAGCAGTCGT	0.016
6	CCCGGTGGTACGAGGTATCTCTAGCGACGAG	0.026
7	CTCGGTGGCGCGAGGTGTATCTATAAACGGC	0.023
8	CTCGGTGGCGCTCTAAGCAGCTGTAGATCGC	0.117
9	CTCGGTGGCGCTCTATGCAACTGGCGACGAG	0.178
10	GACGGTGATATGAGGTATCTCTAGCGTACGC	0.015
11	GACGGTGATATTCTAGGCTTTCGGAAACGAC	0.021
12	GCCGGTCGCGCGAGGCATATCTATAGATCGC	0.027
13	GCCGGTCGCGCTCTAAGCAGCTGTAAACGGC	0.022
14	GTATCGCATATGAAGCACAATCGGAAACGAC	0.078
15	GTATCGCATATGAGGTGTATCTAGAAACGAC	0.041
16	GTATCGCATATGAGGTGTATCTAGCAGTCGT	0.034
17	GTATCGCATATGCGGAGCCGTCAAAATTGGA	0.023
18	GTATCGCATATGCGGCGCCGTCAGAAACGAC	0.055
19	GTATCGCATATTCTAAGCAGCTGTAGATCGC	0.068
20	GTATCGCATATTCTAGGCTTTCGGAAACGAC	0.021
∑		0.97
